# Supplementary material for: The Impact of Vanilla and Lemon Aromas on Sensory Perception in Plant-Based Yogurts Measured with Static and Dynamic Methods
Source: Foods. 2022 Jul 8;11(14):2030. doi: 10.3390/foods11142030 (PMC9319920; doi:10.3390/foods11142030)
Supplement: Supplementary file 1 [file foods-11-02030-s001.zip › foods-1792961-supplementary.pdf]

Table S1. The levels of the main effects (sample, panelist, and session) and interaction effects (sample by session, panelist by sample, and session by panelist) by a three-way ANOVA: A) without the nose clip and B) with the nose clip. Statistically significant differences presented: ***P < 0.001, ** < 0.01, *P < 0.05.

**A)**		**Thickness**		**Stickiness**		**Melting sensation**		**Sweetness**		**Grain-like flavor**		**Aroma intensity**	
	df	F-value	p	F-value	p	F-value	p	F-value	p	F-value	p	F-value	p
Session	3	0.710	0.547	0.642	0.589	1.771	0.154	1.577	0.196	1.452	0.229	0.573	0.633
Sample	4	0.665	0.617	0.984	0.417	1.617	0.172	2.538	0.041*	38.945	<0.001***	31.633	<0.001***
Panelist	9	21.019	<0.001***	11.882	<0.001***	9.038	<0.001***	24.948	<0.001***	6.837	<0.001***	3.785	<0.001***
Sample*Session	12	0.419	0.955	0.520	0.900	0.573	0.862	0.224	0.997	0.208	0.998	0.524	0.897
Panelist*Sample	36	0.784	0.801	0.838	0.728	0.779	0.809	1.592	0.029*	2.361	<0.001***	2.078	<0.001***
Session*Panelist	27	1.157	0.284	1.903	0.008**	1.852	0.010**	2.843	<0.001***	0.491	0.984	0.705	0.857

**B)**		**Thickness**		**Stickiness**		**Melting sensation**		**Sweetness**	
	df	F-value	p	F-value	p	F-value	p	F-value	p
Session	3	1.247	0.294	0.390	0.760	1.723	0.163	0.420	0.739
Sample	4	1.021	0.398	0.189	0.944	0.278	0.892	0.074	0.990
Panelist	9	11.224	<0.001***	16.332	<0.001***	7.262	<0.001***	111.350	<0.001***
Sample*Session	12	0.374	0.971	0.635	0.810	0.291	0.990	0.085	1.000
Panelist*Sample	36	0.621	0.952	0.482	0.994	1.676	0.017*	0.834	0.733
Session*Panelist	27	1.891	0.008	3.114	<0.001***	1.966	0.005**	4.509	<0.001***
